# 

**Published:** 1826-04

**Authors:** 


### Contents

OF

No. 8. Vol. IV. (NEW SERIES) for APRIL, 1826.

[*No. 24, ANALYTICAL SERIES.*]

Art. I.

The History of Ancient and Modern Wines. By A. Henderson, M.D. .. .. .. .. .. .. .. .. .. .. .. 305

II.

Medico-Chirurgical Transactions, Vol. XIII. Part I... .. .. .. 317
Art. 1. Case of Axillary Aneurism successfully treated, by Mr. A. Key .. .. .. .. .. .. .. .. 319
2. Tumour in the Anterior Mediastinum, by A. Gordon, M.D. .. .. .. .. .. .. .. .. 322
3. Injury of the Blood-vessels of the Lower Extremity, by Mr. Chevalier .. .. .. .. .. .. 324
4. Ulceration and Rupture of the Stomach, by Dr. Elliotson .. .. .. .. .. .. .. .. .. 326

(To be continued.)

III.

1. Dr. James Annesley on the Effects of Calomel in Indian Diseases, and on the Mucous Membrane of the Stomach and Bowels .. .. .. .. .. .. .. .. ..
2. An Essay on Mercury, &c. By S. A. Cartwright, M.D. } 328

IV.

Medical Essays. By Marshall Hall, M.D. .. .. .. .. .. 341
1. Essay on Intestinal Irritation .. .. .. .. .. .. 342
2. On the Effects of Loss of Blood.. .. .. .. .. .. 351
3. On Exhaustion .. .. .. .. .. .. .. .. .. .. 356

V.

Mr. Kirby's Additional Observations on severe Forms of Hæmorrhoidal Excrescences; with the History of an Extirpation of the Parotid Gland.. .. .. .. .. .. .. .. .. .. 358

VI.

Dr. Baillie's Posthumous Works .. .. .. .. .. .. .. 364

VII.

An Essay on the Application of Lunar Caustic in certain Wounds and Ulcers. By John Higginbottom, Esq. .. .. .. 379

VIII.

The Study of Medecine. By John Mason Good, M.D. (2d Ed.) 387

IX.

The Lectures of Sir Astley Cooper, Bart. By Mr. Tyrrell .. 397

X.

DROPSY.

1. Dr. Ayre on the Nature and Treatment of Dropsy in the Brain, Chest, &c. .. .. .. .. .. .. ..
2. Dr. Yeats on the Early Symptoms of Water in the Brain, (2d Edition) .. .. .. .. .. .. .. ..
3. Dr. Shearman on the Nature, Causes, and Treatment of Water in the Brain .. .. .. .. .. .. .. 406

XI.

Observations on the Lepra Arabum, or Elephantiasis of the Greeks, as it appears in India. By Whitelaw Ainslie, M.D. &c. 443

XIII.

AUSCULTATION AND THORACIC DISEASES.

1. Dr. Stokes on the Use of the Stethoscope .. .. ..
2. Dr. Bennett on Mediate Auscultation .. .. .. ..
3. Dr. Ryland on the Means of Investigating Diseases of the Chest .. .. .. .. .. .. .. .. .. 449

XIV

Professors Mansfeld, Schenk, and Meyer, on the Antiquity and History of Gastrotomy and Hysterotomy, with successful Cases of Cæsarean Operation .. .. .. .. .. .. 473

XV.

Quarterly Periscope

OF

PRACTICAL MEDICINE;

BEING

*THE SPIRIT OF THE MEDICAL JOURNALS,*

FOREIGN AND DOMESTIC.

I. PHYSIOLOGY.

1. M. Pouillet on Atmospheric Electricity .. .. .. .. 485

2. Dr. Simon's Experiments on Biliary Secretion .. .. .. 488
3. M. Dumeril's Report on Dr. Barry's Experiments on the Venous Circulation .. .. .. .. .. .. .. .. 488
4. Dr. Crowther on Spectral Illusions .. .. .. .. .. 490
5. Dr. Bostock on the State of the Saliva in Mercurialisation .. 490
Reviewer's Observations on Mercurial Action in the System.. 491

II. PATHOLOGY.

6. M. Louis' Researches on Phthisis.. .. .. .. .. .. 492
7. On Angina Pectoris .. .. .. .. .. .. .. .. 496
8. M. Bouillaud on Gangrene of the Lungs .. .. .. .. 498
9. Epilepsy, with remarkable State of the Pulse, by Dr. W. Burnett 502
10. Drs. Bouillaud and Ribes on Inflammation of Veins .. .. 504
11. Remarkable Case of Perforation of the Stomach, in an Adult 516
12. Dr. Whymper on Spontaneous Hydrophobia .. .. .. 518
13. Dr. Troillet on Globus Antiperistalticus .. .. .. .. 519
14. Dr. Gregory's Case of Hæmatoid Tumour of the Brain .. 523
15. M. Scotteten's Case of Hypertrophia Cerebri .. .. .. 523
16. Dr. Bailey on Encysted Formations .. .. .. .. .. 524
17. Inflammation of the Stomach and Liver, simulating Yellow Fever .. .. .. .. .. .. .. .. .. .. 526
18. Mysterious Death, recorded by Lord Clarendon .. .. .. 527

III. SURGERY.

19. Mr. Wardrop's Operation for Carotid Aneurism .. .. .. 528
Remarks on the above Operation .. .. .. .. .. .. 532
20. M. Roux on Staphyloraphia.. .. .. .. .. .. .. .. 533
21. Mr. Bell on Hyoscyamus and Camphor in Gonorrhœa.. .. 535
22. Mr. Shaw on Hæmorrhage after Lithotomy.. .. .. .. 538
23. Mr. Guthrie on Fracture of the Neck and Upper Part of the Femur .. .. .. .. .. .. .. .. .. .. 540
24. Fungus of the Uterus extirpated, by Professor Recamier .. 544
25. Mr. Kirby's Case of Extirpation of the Parotid Gland.. .. 546
26. Mr. Travers on Wounds of the Abdomen, with Cases.. .. 547
Mr. Dix's Case of Wounded Abdomen .. .. .. .. 549
27. Mr. Robart's Case of Urethro-Vaginal Fistula .. .. .. 549
28. M. Ducasse's Case of strangulated Hernia, with Inflammation 550
29. Dr. Begbie's supposed Case of fractured Cervix Femoris .. 551
30. Mr. James on the Removal of Cicatrices .. .. .. .. 553
31. Mr. Oswald's Case of Injury of the Head .. .. .. .. 555

IV. THERAPŒIA.

32. Dr. Hastings on Empyema, with the Reviewer's Remarks .. 557
33. On Laryngeal Phthisis .. .. .. .. .. .. .. .. 566
34. Dr. Granville's Case of complicated Disease.. .. .. .. 566
35. M. Laennec's Hospital Reports—Rheumatism, Pneumonia,&c. 569
36. Dr. Belcher on Neuralgic Amaurosis .. .. .. .. .. 572
37. Dr. Crowther on Vesical Ulceration .. .. .. .. .. 572

*Pharmacology.*

38. On Croton Tiglium Oil, deprived of its Acrid Matters.. .. 573
39. Dr. Darling on Chlorate of Soda .. .. .. .. .. .. .. 574

V. MIDWIFERY.

40. Dr. Pockels on the Evolution of the Human Ovum .. .. 575
41. Successful Case of Cæsarean Section .. .. .. .. .. 578
42. Prolapsed Uterus during Pregnancy .. .. .. .. .. 580
43. Extirpation of a prolapsed and diseased Uterus .. .. .. 580

VI. MEDICAL JURISPRUDENCE.

44. College of Surgeons and its Members .. .. .. .. .. 581
Speeches of Mr. Lawrence, Mr. Tyrrell, Mr. Kingdon, &c. 581-5
Reflexions on these Proceedings .. .. .. .. .. .. 586
Petition of Noah Briggs to Oliver Cromwell's Parliament, for Reform of Abuses in the Colleges .. .. .. .. .. .. 588

XVI.

ANALECTA MINORA; OR MINOR PERISCOPE.

1. Cupping-glasses to poisoned Wounds .. .. .. .. .. .. 589
2. Mr. Pretty on Croup .. .. .. .. .. .. .. .. .. 590
3. Dr. Venables on Milky Blood.. .. .. .. .. .. .. .. 591
4. Dr. Kennedy on Otitis Acuta .. .. .. .. .. .. .. .. 591
5. Dr. Blake on the Trephine in Epileptic Convulsions .. .. 592

XVII.

Bibliographical Record.. .. .. .. .. .. .. .. .. .. 593

XVIII.

INTELLIGENCE, &c.

Regulations respecting Assistant Surgeons in the Navy .. .. 597
Mr. Snell's Gold Obturateurs .. .. .. .. .. .. .. .. 598
Middlesex Infirmary .. .. .. .. .. .. .. .. .. .. 598
Medical Pupil .. .. .. .. .. .. .. .. .. .. .. 598
To those whom it may concern .. .. .. .. .. .. .. .. 599
Mr. Bell's Lectures at the College—a Salamander in the Gallery.. 599

List of Subscribers, &c. .. .. .. .. .. .. .. .. 600

